# Relationship Between Gender and Clinician’s Subjective Experience during the Interaction with Psychiatric Patients

**DOI:** 10.2174/1745017902117010190

**Published:** 2021-12-22

**Authors:** Federico Dazzi, Laura Fonzi, Mauro Pallagrosi, Marina Duro, Massimo Biondi, Angelo Picardi

**Affiliations:** 1 Department of Human Sciences, LUMSA University, Rome, Italy; 2 Italian Psychoanalytic Society, Rome, Italy; 3 Department of Human Neuroscience, Sapienza University of Rome, Rome, Italy; 4 Centre for Behavioural Sciences and Mental Health, Italian National Institute of Health, Rome, Italy

**Keywords:** Gender, Clinician’s subjective experience, ACSE, BPRS, Psychiatric diagnosis, Questionnaire

## Abstract

**Introduction:**

The clinician’s subjective experience can be a valuable element for diagnosis and treatment. A few factors have been recognized that affect it, such as the patient’s personality, the severity of psychopathology, and diagnosis. Other factors, such as patient’s and clinician’s gender, have not been specifically investigated. The aim of this study is to explore the impact of gender differences on the clinician’s subjective experience in a large sample of psychiatric patients.

**Methods:**

The study involved 61 psychiatrists and 960 patients attending several inpatient and outpatient psychiatric settings. The clinicians completed the Assessment of Clinician's Subjective Experience (ACSE) questionnaire after observing each patient for the first time.

**Results:**

In multivariate analysis, higher scores on the Difficulty in Attunement (p < 0.001), Engagement (p<0.05), and Impotence (p<0.01) scales were significantly associated with female clinician gender, whereas higher scores on the Tension and Disconfirmation scales were significantly associated with male clinician gender. The scores on all ACSE dimensions were also associated with the severity of psychopathology.

**Conclusion:**

The findings suggest that clinician’s gender might affect a clinician’s emotional response toward patients. Specific attention to this issue might be useful in clinical situations, not only in terms of promoting gender-balanced teams but also in terms of enhancing self-observation in clinicians evaluating patients for the first time.

## 1 INTRODUCTION

The lived experience of the clinical encounter, as it is subjectively perceived by both participants, is a domain that received scarce explicit attention in psychiatric empirical research. The clinician’s subjective experience, in particular, is largely neglected, as contemporary psychiatry tends to disregard its contribution to both diagnostic formulation and treatment outcome.

On the contrary, according to the classical psychopathological tradition, the clinician’s personal sensitivity is a crucial clinical skill, especially during the diagnostic process [1]. Prominent scholars like Binswanger, Minkowski, Rümke and Tellenbach [2-5] used to consider the perception of intersubjective ruptures and dissonances emerging during the interaction as an essential guide for both diagnostic reasoning and therapeutic intervention [6-8].

In the psychotherapeutic field, the clinician’s subjectivity still keeps its centrality, and it is considered as an important factor affecting the patient’s evaluation, the therapeutic alliance, and the effectiveness of treatment. This consideration especially stems from the studies about *countertransference*, which, in its current meaning, encompasses all the conscious and unconscious feelings and reactions that can arise in a clinician when he or she is in a relationship with a patient [9-11].

In fact, there is substantial literature about the factors affecting the clinician’s subjective experience within psychotherapeutic settings. It mainly relies upon two assessment tools, the *Therapist Response Questionnaire* (TRQ) [12] and the *Feeling Word Checklist* (FWC) [13]. Overall, these studies investigated the association between the therapist’s subjective experience and both the patient’s (*e.g*., symptoms, personality, diagnosis) and the therapist’s (*e.g*., theoretical background, years of experience) characteristics, and they reported some consistent patterns. For instance, a patient’s personality was found to significantly influence the clinician’s feelings in many studies [12, 14-19]. Some evidence has also been provided concerning the patient’s psychiatric symptoms or diagnosis [14, 20, 21], while no therapist’s characteristic has been found to be related to his or her countertransference reaction.

Conversely, the investigations about the clinician’s subjective experience in psychiatric settings are fewer and scattered. Most studies generically examined the emotional reactions of all staff members [22-24], with a few studies focusing on psychiatrists only. A group of researchers used a short version of the TRQ to explore the psychiatrist’s emotional reaction to patients who attempted suicide or were at high risk for suicide [25, 26]. Other studies used an instrument specifically developed to explore the psychiatrist’s subjective experience during the diagnostic evaluation, *i.e*., the *Assessment of Clinician’s Subjective Experience* (ACSE) [27], to investigate its relationship with the patient’s categorical [28] and dimensional [29, 30] diagnosis. These studies provided some empirical support to the conceptualizations put forth by classical psychopathologists about the relevance of “diagnostic feeling.”

The empirical studies about the clinician’s feelings considered gender only as an ancillary factor. In a few of them, nonetheless, the clinician’s and patient’s gender showed interesting relationships with the clinician’s emotional reaction. In two studies using the TRQ, for example, male clinicians scored significantly higher than female ones in the “criticized/mistreated,” “overwhelming”, “sexualized” [21], and “aroused/reacting” [31] scales, while female clinicians obtained higher scores in the “fulfilled/engaging” scale [31]. A few studies on staff members working with psychiatric patients also showed some significant differences regarding patient’s gender. In one study about the staff-patient relationship in outpatient settings, staff members reported higher levels in the “rejecting,” “unhelpful,” and “controlled” scales of the FWC with female patients [32], while in a study performed in acute inpatient units staff members experienced more intense “on guard” and “inadequate” feelings and less intense “overwhelming” feelings when facing male patients [33].

Thus, it seems that gender may significantly affect the clinician’s subjective experience, such that it may have a considerable influence on the clinician-patient interaction in terms of both therapeutic alliance and diagnostic reasoning.

However, to our knowledge, only three empirical studies explicitly addressed the issue of gender differences within clinical settings. De Vogel & Louppen [34] investigated the feelings of staff members toward their most complex patients in a forensic psychiatric hospital. They reported a higher level of positive feelings toward women, whereas male patients evoked more negative feelings. Female staff members felt more accepting, helpful, receptive, affectionate, strong, relaxed toward female patients, and more threatened and overwhelmed with male patients. Male staff members felt instead more angry, anxious, unhelpful, but also sympathetic, and less receptive toward male patients. Some differences were observed between male and female staff members. Compared to women, men were more cautious and embarrassed (in many cases because of the risk of being falsely accused of sexually inappropriate behaviour) and less motherly when working with female patients, whereas they felt more relaxed with male patients. Latts & Gelso [35] reported that male therapists were more likely to provide avoidance responses to rape survivors, but this finding was not later confirmed by Eizirik *et al*. [36], who did not report significant differences in countertransference feelings between male and female therapists treating women victims of sexual violence.

The aim of this study is to explore the impact of gender on the psychiatrist’s subjective experience more extensively during everyday clinical practice in order to better understand its role during the development of the clinician-patient relationship. Our hypothesis is that both clinician’s and patient’s gender may affect the clinician’s way of feeling towards patients, as some previous findings seem to suggest.

## 2 METHODS

### 2.1 Setting and Participants

The study was performed in a number of psychiatric inpatient (psychiatric intensive care units and the emergency departments) and outpatient units of the National Health Service in Rome, Italy. For every clinical and diagnostic assessment of a previously unknown patient, clinicians working at these units were asked to complete the Assessment of Clinician’s Subjective Experience (ACSE) instrument [27] and the Brief Psychiatric Rating Scale – Expanded Version (BPRS-E) [37-39] at the end of each visit for eligible patients. Patients were included if they were 18 years old and Italian (to rule out potential problems in mutual understanding due to language difficulties in foreign patients), whereas they were excluded if they had one of the following diagnoses: significant cognitive impairment, mental retardation, substance use disorder or major non-psychiatric medical illness (since, according to the organization of psychiatric services in Italy, they are usually treated by other psychiatric and even non-psychiatric services).

Overall, 61 psychiatrists were involved in the study. They had different theoretical backgrounds and levels of experience, with 23 senior psychiatry residents and 38 psychiatrists with a mean of post-residency experience of 10.7±5.0 years. The mean number of patients assessed per clinician was 12.7±11.6 (range 1-40). The mean duration of the visit was 40.9±15.5 minutes. The characteristics of the clinicians are reported in Table **1**. They recruited a total of 960 patients, of whom 44.6% were males and 55.2% were females; 43.2% were evaluated in outpatient clinics and 56.8% in hospital settings; 42.0% were seen by a male psychiatrist and 58.0% by a female psychiatrist. Patients’ clinical and demographic characteristics are reported in Table **1**.

### 2.2 Assessment

For each patient, the following demographic and clinical information was collected through a standardized form: age, sex, education, diagnosis and BPRS-E total score.

The ACSE is a self-rated instrument specifically developed to measure clinicians’ subjective experience toward patients [27, 40]. It consists of 46 items, each rated on a 5-point scale ranging from 0 to 4, gathered into 5 factorially derived scales [27], named Tension, Difficulty in Attunement, Engagement, Disconfirmation, and Impotence.

The Tension scale (range 0-44) measures physical tension and clumsiness, reduced spontaneity, and feelings of worry, nervousness, and alarm; the Difficulty in Attunement scale (range 0-40) describes difficulty in establishing emotional contact, being empathic, understanding the patient's experience, and communicating with the patient; the Engagement scale (range 0-32) describes the degree of involvement with the patient, including feelings of boredom, indifference, detachment, lack of attention and, conversely, desire to take care of the patient, and feelings of deep involvement in the patient-physician relationship, emotional closeness and tenderness; the Disconfirmation scale (range 0-36) includes items indicating a failure to establish an authentic relationship with the patient, and feelings of being manipulated, rejected, criticized or devalued by the patient; the Impotence scale (range 0-32) consists of items describing feelings of helplessness, frustration, desolation, emptiness, loneliness, and being drained. For each scale, higher scores reflect the greater intensity of the measured dimension.

The BPRS-E is a widely used 24 item clinician-rated scale assessing a range of symptoms across different psychopathological domains (negative affect, positive symptoms, negative symptoms, activation, and disorganization), with the total score representing a measure of the overall severity of psychopathology. In the expanded version [38, 39], which includes 24 items scoring from 1 (not present) to 7 (extremely severe), the authors provided a manual of administration with defined anchor points, detailed probe questions, and rules for scoring. In the current study, we used the Italian version and the 0-6 scoring system, with the total score ranging from 0 to 144.

### 2.3 Statistical Analyses

All statistical analyses were carried out using SPSS for Mac, version 22.0 (SPSS Inc., Chicago, Ill., USA). All tests were two-tailed, with alpha set at 0.05. First, we performed descriptive analysis using appropriate descriptive statistics, *i.e*., mean and standard deviation or frequencies. Then, we examined associations between the clinician’s experience and patient gender by performing univariate analysis with Student’s *t*-test to examine differences in mean scores on the ACSE dimensions between male and female patients. Subsequently, we used general linear model analysis to test for differences in ACSE scores by patient gender while controlling for clinician’s gender and symptom severity as measured by the BPRS total score. We also tested for the interaction between patients’ and clinicians’ gender.

## 3 RESULTS

As shown in Table **2**, both male and female clinicians reported significantly higher levels of Tension and Difficulty in Attunement with male patients as compared with female patients, while only the female clinicians scored significantly higher on the Impotence scale with male patients as compared with female patients.

In multivariate analysis, higher scores on the Tension scale were found to be significantly associated with higher BPRS total scores (p<0.001), male patient gender (p<0.001), and male clinician gender (p<0.05). Higher scores on the Difficulty in Attunement scale were found to be significantly associated with higher BPRS total scores (p<0.001) and female clinician gender (p<0.001). Higher scores on the Engagement scale were found to be significantly associated with female clinician gender (p<0.05), with a non-significant interaction between clinician and patient gender (p=0.13). Higher scores on the Disconfirmation scale were found to be significantly associated with higher BPRS total scores (p<0.001) and male clinician gender (p<0.05). Finally, higher scores on the Impotence scale were found to be significantly associated with higher BPRS total scores (p<0.001) and female clinician gender (p<0.01), with a weak tendency towards an interaction between clinician and patient gender (p=0.10).

## 4 DISCUSSION

The present study provides empirical support for the hypothesis that gender affects the psychiatrist’s subjective experience during the clinical encounter in everyday clinical practice. This seems to be true for both patient’s and clinician’s gender.

With regards to patient’s gender, our main finding is that the encounters with male patients are associated with higher scores on the ACSE Tension scale as compared to those with female patients. The observation concerns both male and female clinicians, and the multivariate analysis shows that it is not fully explained by differences in clinical severity. This result is consistent with previous observations that staff members and clinicians are more likely to feel threatened, overwhelmed [34], and on guard [33] when facing male patients than female patients, regardless of their diagnosis.

In previous studies, this finding was explained mainly with the idea that women are generally perceived as less threatening than men due to their lesser physical strength. However, we hypothesize that, beyond the concrete bodily difference, the peculiar gender-related expression of aggressiveness may have a role. Indeed, it is generally accepted that men are more likely to express aggressive behaviour overtly, whereas aggressiveness is more commonly indirect and “relational” among women [41, 42]. A higher intensity of feelings like tension, alertness, and stiffness might then reflect an instinctual heightened perception of a forthcoming aggressive outburst from male patients, as compared to female ones. Interestingly, this sort of natural “preconception” is not confirmed by epidemiological data and may represent a clinically significant perceptual bias. Indeed, a number of authors [43, 44] observed that violent behaviour is as likely among female as male psychiatric patients [43, 44]. Also, even though men can cause more severe injuries [45], it was pointed out that psychiatrists risk underestimating the possibility of violence among female patients [46].

Thus, clinicians should keep in mind the risk of a “gender-biased” increased sense of danger when encountering male patients, and they should carefully consider both the risk of overestimating the chance of male violence and the influence that this perception may have on the quality of the diagnostic evaluation and the development of a good therapeutic alliance.

Conversely, patient’s gender does not seem to affect the other dimensions of the clinician’s subjective experience. Conflicting evidence has been previously reported in this regard. On the one hand, a recent study on suicidal patients [26] showed that the patient’s gender does not affect the emotional response of young psychiatrists in terms of the TRQ dimensions of Affiliation, Distress, and Hopefulness. On the other hand, two studies on staff members’ feelings found that female patients can elicit either more negative [32] or more positive [34] feelings than male ones. Overall, it seems that subjective experiences such as attunement, engagement, alliance, or frustration are not strongly affected by the patient’s gender, both in its biological and cultural aspects, with other elements, such as the psychopathological ones playing a more relevant role [28, 29].

Concerning clinician’s gender, it showed a more articulated profile of associations with the ACSE scales than patient’s gender. Female clinicians obtained higher scores than male ones on the Difficulty in Attunement, Engagement, and Impotence scales, while the opposite occurred for Tension and Disconfirmation. Only small tendential correlations were detected for specific gender matches.

With the literature still providing a poorly defined picture on this topic, our findings are only partially consistent with previous research. A few studies reported no significant differences in countertransference feelings between male and female clinicians [18, 32, 36], whereas other studies described findings similar to ours. Indeed the higher level of Tension and Disconfirmation found in male clinicians seems to be consistent, according to our interpretation, with previous observation of male clinicians feeling more criticized/mistreated [21], aroused/reacting [31], angry, anxious, and unhelpful [34]. Also, the higher level of engagement found in female clinicians is consistent with a previous study in which female clinicians scored higher in the TRQ “fulfilled/ engaging” scale [31]. On the contrary, to our knowledge, no previous studies explicitly investigated gender differences in Difficulty in Attunement and Impotence, which appear to be a novel finding that is worthy of further investigation.

Even though the lack of specific literature on this topic makes it difficult to draw comparisons with previous research, it might be useful to interpret our findings through the lens of some psychological and cultural considerations. In our sample, male clinicians exhibited more intense feelings of tension, alertness, anger and perception of being judged, rejected, or manipulated, as they suffered from the interaction with patients, especially in the interpersonal dimensions of conflict and role recognition. Such findings are consistent with a theoretical and empirical tradition about the issues of men’s greater proneness towards aggressiveness, confrontational attitude, and self-affirmation importance [42, 47]. According to this perspective, the male clinicians’ heightened perception of being devalued and challenged on identity and professional authority might be more evident, especially within psychiatric settings, where a patient’s rejecting and provocative disposition is not uncommon.

Naturally, as the ACSE is a self-report questionnaire exploring the clinician’s lived experience and not his behaviour, the prominence of such feelings does not imply that male clinicians behave according to them. However, it is interesting that a few studies about the doctor-patient relationship in medical settings showed that male physicians are more prone than female ones to be assertive and oriented to technical aspects during the visits [48, 49]. Likewise, a study on first diagnostic consultations in psychiatry found a greater focus on patient’s symptomatic concerns and less receptivity to “not strictly clinical” cues in male clinicians than in female ones [50]. In other words, provided that it is an inevitable oversimplification, we hypothesize that male clinicians are particularly sensitive to the loosening of the professional asymmetry and the activation of antagonistic patterns when encountering patients and that being aware of this sensitivity could help them, especially during the early stages of the acquaintance.

As far as female clinicians are concerned, they showed more intense feelings of empathic failure, anguish, solitude and impotence, together with the perception of being close and emotionally involved with patients. These findings, too, can be read in the light of some general gender characteristics. With regard to empathic understanding, the psychological literature traditionally attributed to women a better capacity to be empathic and sensitive to the other’s mental and emotional state [51, 52], as well as a more engaged and warmer attitude than men [50, 53]. Some studies carried out in medical and psychiatric settings seem to support this view, showing a more receptive disposition towards patients’ emotional expressions and a more talkative style in female clinicians [50, 54]. On the contrary, two more recent studies [55, 56] reported negligible differential performance in experimental empathy tasks between men and women. It is worth noting that research in this field might be biased by role stereotypes, especially when performed through self-reports. Indeed, a study conducted by Baez *et al*. [56] (to which we refer for a thorough review of this topic) highlighted the influence of gender-role stereotypes, as it showed that while gender differences could be observed through self-reports, such differences were negligible when performing experimental empathy tasks. Extending this consideration to our results, it is possible that the differences we observed were affected by gender-role stereotype effects.

In essence, it seems that female clinicians tend to engage themselves in closer clinical relationships, even though it does not imply that they are more accurate in empathically understanding the patients. After all, empathic comprehension and sympathetic involvement are considered as different experiential dimensions by a solid philosophical tradition [57]. Our finding that female clinicians reported higher levels of engagement, which is more similar to the experience of sympathy, while they scored higher in Difficulty in Attunement, which pertains to the empathy domain, suggests that female psychiatrists, paying more attention to their empathic and emotional engagement, could more readily feel a lively involvement with the patients themselves and be more sensitive to the ruptures in the empathic connection. This may also account for the observed gender differences in the Impotence dimension.

### 4.1 Limitations

This study covered different types of settings (both outpatient and inpatient services), included a large number of patients, and involved numerous clinicians with very different theoretical backgrounds and degrees of experience, which minimised the risk of biases related to specific expertise and increased the generalizability of the findings. Nevertheless, some limitations must be acknowledged.

First, although in the analysis we adjusted for the patient’s psychopathological severity (BPRS-E total score), we did not take into account other clinical factors that may have confounded the effect of gender. We know, in particular, that patient’s personality and psychiatric diagnosis may affect the clinician’s subjective experience. However, fully disentangling personality traits from gender attitudes is difficult and may represent mainly an abstract issue since the two aspects are often intertwined. Gender is not only a biological issue, after all. Concerning diagnosis, we could not perform a stratified analysis for broad diagnostic categories as it would have significantly reduced statistical power. However, adjusting for the BPRS-E total score should have reduced the risk of diagnostic bias since psychopathological severity generally enables a plausible, though the rough, distinction between clinical macro-categories (*e.g*., anxious *vs*. psychotic patients).

Also, it should be recognised that any study that involves gender as a variable deals with a multifaceted issue. Gender is not only a biological matter, as it also encompasses socio-cultural aspects, such as stereotypes, prejudices, gender-related expectations and experiences, *etc*., that might affect the results, especially, as discussed above, when collected through self-reports. As we did not take this issue into account in the methodological design, our findings cannot disentangle the cultural and biological influences, preventing us from weighting these aspects. However, the clinicians were not informed of the aims of the study, and this should have mitigated the effect of gender prejudices and stereotypes.

## CONCLUSION

Although the limitations described above suggest caution in interpreting our findings, our study suggests that gender, with all its stratified complexity, seems to have an impact on the clinician’s subjective perception of the patient. The results indicate that both patient’s and clinician’s gender may affect the intersubjective atmosphere of the clinical encounter in everyday psychiatric settings. This finding is consistent with some previous studies and broadens the scope of their results as it concerns a large sample of clinician-patient interactions.

The tentative evidence of a role of gender in influencing, even implicitly, the psychiatrist’s instinctual perceptions during patient’s assessment further underscores the importance, for clinicians, of carefully observing their natural reactions. Some immediate and pre-reflective responses, in fact, may contribute to the risk of evaluation biases (*e.g*., in predicting aggressive outbursts) and difficulties in developing a therapeutic alliance.

Also, our results encourage a reflection about the organization of psychiatric services and the potential advantage of building gender-balanced teams. Indeed, although psychiatric care is not a matter of gender, it is plausible that in some cases, the choice of a “gender-matched” clinician can help address the patient’s peculiar needs (*e.g*., a provocative and deprecating patient may be initially better engaged by a female clinician).

Further research is needed to address the limitations of this study, especially to better disentangle the role played by biological and socio-cultural factors.

## Figures and Tables

**Table 1:** Clinicians’ and patients’ characteristics.

-	**Clinicians (N=61)**	
**Dependent Variable**	**N (%)**	**Mean ± SD**
**Sex**		
Male	23 (37.7)	
Female	38 (62.3)	
**Age**		37.9 **±**7.5
**Years of Post-Graduation Experience**		10.8 **±** 6.8
**Theoretical background**		
Psychodynamic theories	24 (39.3)	
Clinical/biological psychiatry	18 (29.5)	
Cognitive-behavioral theories	11 (18.0)	
Phenomenology	4 (6.6)	
Family systems theory	2 (3.3)	
Transactional theory	2 (3.3)	
	**Patients (N=958)**	
**Sex**		
Male	428 (44.6)	
Female	530 (55.2)	
**Age**		42.8 ± 15.1
**Education**		
Less than completed primary	18 (1.9)	
Primary school	36 (3.8)	
Junior high school	219 (22.8)	
Senior high school	450 (46.9)	
University degree	213 (22.2)	
**Primary Axis I Diagnosis**		
Schizophrenia	185 (19.3)	
Acute psychosis	37 (3.8)	
Schizoaffective Disorder	43 (4.5)	
Delusional Disorder	29 (3.0)	
Unipolar Depression	149 (15.5)	
Bipolar Disorder, manic, hypomanic, or mixed episode	91 (9.5)	
Bipolar Disorder, depressive episode	18 (1.9)	
Bipolar Disorder, unspecified	34 (3.5)	
Dysthymic Disorder	21 (2.2)	
Other Mood Disorders	51 (5.3)	
Anxiety Disorder	89 (9.3)	
Obsessive-Compulsive Disorder	18 (1.9)	
Eating Disorder	54 (5.6)	
Somatic Symptom Disorder	9 (0.9)	
Adjustment Disorder	22 (2.3)	
Other disorders	18 (1.9)	
No Axis I diagnosis	91 (9.5)	
**Primary Axis II Dagnosis**		
Cluster A Personality Disorder	42 (4.4)	
Cluster B Personality Disorder	187 (19.5)	
Cluster C Personality Disorder	58 (6.0)	
Personality Disorder, not otherwise specified	32 (3.3)	
No Axis II Diagnosis	641 (66.8)	
**BPRS total score**		49.7 **±** 15.4

The number of patients may not add to 960 and 100% due to a few missing data.

**Table 2:** ACSE scores (mean±SD) by patient gender.

-	**Male Clinicians**		**Female Clinicians**	
**Patient Gender**	**Male (N=205)**	**Female (N=195)**	**Male (N=229)**	**Female (N=321)**
**ACSE Scores**				
Tension	9.8±6.7***	7.3±6.2	8.4±6.7***	5.6±5.2
Difficulty in Attunement	16.8±8.3**	14.7±7.8	16.5±7.9*	15.1±7.6
Engagement	16.6±4.9	17.3±4.7	17.9±5.1	17.5±4.7
Disconfirmation	8.6±6.0	8.5±6.3	7.2±5.9	7.2±5.2
Impotence	7.8±5.4	7.5±5.8	8.6±6.4**	7.2±5.8

*** p<0.001; ** p<0.01; * p<0.05. Numbers may not add to 960 due to a few missing data.

## Data Availability

The data that support the findings of this study are available from the corresponding author [A.P] upon reasonable request. The data are not publicly available due to legal restrictions, as they contain information that could compromise the privacy of research participants.

## LIST OF ABBREVIATIONS

TRQ: = Therapist Response Questionnaire
FWC: = Feeling Word Checklist
ACSE: = Assessment of Clinician’s Subjective Experience
BPRS: = Brief Psychiatric Rating Scale
